# Lentiviral Transgenic MicroRNA-Based shRNA Suppressed Mouse Cytochromosome P450 3A (CYP3A) Expression in a Dose-Dependent and Inheritable Manner

**DOI:** 10.1371/journal.pone.0030560

**Published:** 2012-01-24

**Authors:** Yong Wang, Hai-Hong Hu, Hao Pang, Xiao-Yang Zhou, Lu-Shan Yu, Lu-Lu Wang, Cang'e Liu, Ke-Nan Guo, Cong Zhao, Qin Liu, Ben-Hua Zeng, Huan Tang, Hai-Tao Shang, Su Zeng, Hong Wei

**Affiliations:** 1 Department of Laboratory Animal Science, College of Basic Medical Sciences, Third Military Medical University, Chongqing, China; 2 College of Pharmaceutical Sciences, Zhejiang University, Hangzhou, China; 3 School of Animal Science and Technology, Southwest University, Chongqing, China; Istituto Dermopatico dell'Immacolata, Italy

## Abstract

Cytochomosome P450 enzymes (CYP) are heme-containing monooxygenases responsible for oxidative metabolism of many exogenous and endogenous compounds including drugs. The species difference of CYP limits the extent to which data obtained from animals can be translated to humans in pharmacodynamics or pharmacokinetics studies. Transgenic expression of human CYP in animals lacking or with largely reduced endogenous CYP counterparts is recognized as an ideal strategy to correct CYP species difference. CYP3A is the most abundant CYP subfamily both in human and mammals. In this study, we designed a microRNA-based shRNA (miR-shRNA) simultaneously targeting four members of mouse CYP3A subfamily (CYP3A11, CYP3A16, CYP3A41 and CYP3A44), and transgenic mice expressing the designed miR-shRNA were generated by lentiviral transgenesis. Results showed that the CYP3A expression level in transgenic mice was markedly reduced compared to that in wild type or unrelated miR-shRNA transgenic mice, and was inversely correlated to the miR-shRNA expression level. The CYP3A expression levels in transgenic offspring of different generations were also remarkably lower compared to those of controls, and moreover the inhibition rate of CYP3A expression remained comparable over generations. The ratio of the targeted CYP3A transcriptional levels was comparable between knockdown and control mice of the same gender as detected by RT-PCR DGGE analysis. These data suggested that transgenic miR-shRNA suppressed CYP3A expression in a dose-dependent and inheritable manner, and transcriptional levels of the targeted CYP3As were suppressed to a similar extent. The observed knockdown efficacy was further confirmed by enzymatic activity analysis, and data showed that CYP3A activities in transgenic mice were markedly reduced compared to those in wild-type or unrelated miR-shRNA transgenic controls (1.11±0.71 vs 5.85±1.74, 5.9±2.4; P<0.01). This work laid down a foundation to further knock down the remaining murine CYP3As or CYPs of other subfamilies, and a basis to generate CYP knockdown animals of other species.

## Introduction

RNA interference (RNAi) is a post transcriptional gene silencing mechanism, which is conserved among a broad variety of eukaryotic organisms including mammalian species [1]–[4]. Mammalian models specifically lacking target gene expression are powerful tools for deciphering gene functions and generating bio-medical models. Traditional method for generating gene-disrupted animals is through ES cell-based homologous recombination. Although this method is effective, it is limited by the low efficiency of DNA homologous recombination, time-consuming and labor-intensive cross strategies for obtaining homozygous mutant individuals, and more importantly, the lack of ES cells derived from other mammalian species limits its application to other important mammalian model animals such as rats, pigs and monkeys. Since RNAi is a highly conserved gene silence mechanism, RNAi provides an alternative method for specifically disrupting mammalian gene expression on both cell and individual level.

By transgenic expression of shRNA molecules, mammalian endogenous gene expression can be specifically inhibited in vivo [5]–[9]. Recently, a microRNA-based shRNA (miR-shRNA) system has been developed and utilized to effectively knock down target gene expression constitutively or conditionally [10]–[12]. Compared to conventional shRNA molecules, gene silence mediated by miRNA-shRNA has advantages. First, it has higher efficacy in knock-down of target gene expression, for it works through the existing natural mechanisms or pathways in cells which are used by endogenous miRNA molecules [10]. Second, its expression can be driven by polymerase II promoter, as that for endogenous miRNA molecules, which rendered the gene silence mediated by RNAi to be more controllable.

Mammalian animals, such as mice, are extensively used models for the studies of pharmacokinetics or pharmacodynamics in drug development. However, the remarkable species differences in biological activity of cyptochromosme P450 (CYP) enzymes limits the extent to which data obtained from animals can be translated to humans [13]–[18]. CYP enzymes are heme-containing monooxygenases responsible for the oxidative metabolism of many endogenous and xenobiotic compounds, which play critical roles in drug metabolism and are closely related to toxicity or inefficacy of drugs [19]–[21]. Transgenic expression of human CYP enzymes in animals has been recognized as an effective method to correct the inter-specie disparity of CYP enzymatic activity [16], [18], [22], however the counterparts of animal endogenous CYPs usually show extensive overlaps in substrate specificity and tissue distribution compared to that of human [22], [23]. Therefore, transgenic expression of a human CYP in a background lacking or with largely reduced expression of the orthologous counterparts of animal endogenous CYP enzymes is considered to be an ideal strategy to analyze its involvement in metabolism of target drugs, or its correlation to drug toxicity or inefficacy [23].

Human cytochromose P450 3A (CYP3A), a subfamily of cytochromosme P450 enzymes, is involved into the metabolism of more than 50% of clinically available drugs, and exhibits extensive overlaps in enzymatic activity compared to the orthologous counterparts of mammalian animals. Therefore, transgenic expression of human CYP3A in animals lacking the expression of endogenous counterparts is of great value for drug development [23], [24]. Recently, a knock-out mouse lacking all the muring CYP3A genes was generated by ES cell-based DNA homologous recombination [23]. However, the CYP enzyme is a superfamily consisting of many subfamilies and dozens of members, it would be a highly costly, time-consuming and labor-intensive process to knock-out CYP genes one by one though ES cell-based technology. And beside, the ES cell-based technology is currently not applicable to other mammalian species of great importance in pharmacokinetics or pharmacodynamics studies, such as rat, dog and monkey, mainly due to the lack of established ES cell lines derived from these species. In contrast, since RNAi is a conserved mechanism among eukaryotic organisms, the RNAi-based gene knock-down technology, which is at least theoretically applicable to all mammalian species, provides an alternative method to disrupt endogenous CYP enzyme expression.

This study was intended to try to knock down mammalian endogenous CYP3A enzyme expression by RANi-based technology in vivo using a miR-shRNA system, and thereby to find a simple, effective and general method to generate mammalian models lacking or with largely reduced endogenous CYP enzyme expression. In this study, using a designed miR-shRNA simultaneously targeting four members of CYP3A, knock-down mice with markedly reduced expression of CYP3A, the most abundant CYP enzyme subfamily in mice which is considered to be the orthologous counterpart of human CYP3A, was generated by RNAi-based technology. We found that the transgenic miR-shRNA targeting murine CYP3A, which was delivered by lentiviral vector, suppressed CYP3A expression in a dose-dependant and inheritable manner in mice. This work laid down a foundation to further knock down other murine CYP3A genes or other CYP subfamily genes, and also provide a basis to generate CYP gene knock-down animals of other mammalian species of biomedical importance.

## Materials and Methods

### Animals

Mice of FVBN inbred strain were used in this study, which were purchased from SLAC Laboratory Animal Co.,Ltd (Shanghai, China) and maintained under specific pathogen-free conditions in Laboratory Animal Centre of our university. All the protocols involving the use of animals were approved by the Institutional Animal Care and Use Committee of Third Military Medical University (Approval ID: SYXK-PLA-2007036).

### Vector design and transgenic mouse production

The miR-shRNA sequences targeting CYP3A11, CYP3A16, CYP3A41 and CYP3A44 were designed using the on-line RNAi design algorithm at http://katahdin.cshl.org:9331_siRNA_RNAi.cgi?type_shRNA as previously described [10]. To place the shRNA sequences into miR30 context, a 97-mer sequence containing the designed shRNA was retrieved through the RNAi design algorithm, which was then subcloned into the site of pri-miRNA area downstream the eGFP coding sequence (CDS) in pRIME vector as previously described [10]. Then, the eGFP-miRNA fragment was excised from the recombinant pRIME vector and subcloned into the lentiviral vector FUW, and the resulted recombinant vector was named FUW-eGFP-miR-shRNA in this article.

To test the efficacy of designed shRNA sequences, lentiviral vectors were packaged into lentiviral particles as previously described [25] and infected primarily cultured mouse hepatic cells derived from an adult female mouse. Each infection had three duplicates. At 48 h post infection, using CellAmp™ Whole Transcriptome Amplification Kit (Takara, Dalian, China), cDNA samples were prepared from infected or uninfected hepatic cells as well. A pair of degenerate real-time PCR primers complementary to mRNA sequences of CYP3A11, CYP3A44 and CYP3A41 was designed, of which the sequences were 5′-CTCAATGGTGTGTATATCCCC-3′ (forward) and 5′-GATGTTCTTAGACACTGCC-3′ (reverse) respectively. Using the prepared cDNA as templates, the expression of CYP3A and the internal control gene Rps18 were simultaneously detected in one PCR system. The mixed PCR products were subjected to gel electrophoresis, and semi-quantitation of CYP3A expression was obtained by comparing the band density of the PCR product for CYP3A and that for Rps 18 using the software Gel Pro.4.0. The primers for detecting Rps18 were 5′-AAATAGCCTTCGCCATCAC-3′ (forward) and 5′-TCACTCGCTCCACCTCATC-3′ (reverse) respectively. The two primer pairs for CYP3A and Rps 18 both corresponded to different exons, and the sizes of PCR products were 421 and 129 bp respectively. The PCR reaction conditions were: 95°C 5 min; then entered the circle: 94°C 30 s, 54°C 30 s, 72°C 30 s; run 30 more circles, and then 72°C 8 min.

The shRNA with higher efficacy was selected to generate transgenic mice using FVBN mice by lentiviral transgenesis as previously described [26], [27]. Transgenic founder mice were screened by PCR using genomic DNAs as templates. The sequences of the primer pair for founder mouse screen were 5′-GCGGATCCTACCGGTCGCCACCATGGTGAGCAA-3′ (forward) and 5′-GCGGCGCGCCCAATTGAAAAAAGTGATTTAATTTATACC-3′ (reverse) respectively, and the size of PCR product was 1012 bp. The PCR conditions were: 95°C 5 min; then entered the circle: 94°C 30 s, 60°C 30 s, 72°C 1 min; run 30 more circles; then 72°C 5 min.

### Real-time RT-PCR

Total RNA samples were prepared from livers of adult mice at 8-week age, treated with DNaseI and reversely transcribed as previously described [27]. To detect CYP3A expression and that of the internal control Rps18 quantitatively, the resulted cDNA samples were subjected to real-time PCR using the same primer pairs as mentioned above. The real-time PCR reaction systems for both CYP3A and Rps 18 included: PCR primers (10 µM) 1 µL each; SYBR® Premix Ex Taq™ II(2×)(Cat No.:DRR081A; Takara, Dalian, China) 12.5 µL; cDNA preparation 2 µL; ddH_2_O added up to 25 µL. The PCR conditions were: 95°C 3 min; then entered cycles: 94°C 5 s, 60°C 30 s, plate read; run 39 more cycles; then 72°C 3 min, 95°C 1 min, 60°C 1 min.

The relative expression of CYP3A was calculated using Pfaffl equation: Ratio = (E_target_)^ΔCt(target)^/(E_ref_)^ΔCt(ref)^, where Ratio stands for the proportions of CYP3A expression of transgenic mice to that of wild-type mice, E_target_ for the E value (amplification efficiency) of real-time PCR for CYP3A, E_ref_ for the E value for internal control (Rps 18) and ΔCt for the Ct difference value between the Ct values for CYP3A(target) or Rps18(Ref) of transgenic and wild-type mice.

To quantitatively detect the expression level of miR-shRNA molecule in transgenic mice, using the same reversely transcribed total RNA samples as above, real-time PCR was performed with another primer pair corresponding to eGFP CDS and pri-miRNA area of the transgene construct respectively. The primer sequences were 5′-CTACCTGAGCACCCAGTCCG-3′ (forward) and 5′-TCCCAGCAAGTGTTTCCAAGAT-3′ (reverse) respectively, and the size of PCR product was 233 bp. The real-time PCR system and reaction conditions for miR-shRNA were the same as that for CYP3A, except that the melting temperature was 55°C. To determine the copy numbers of miR-shRNA transcripts in samples, the FUW-eGFP-miR-shRNA plasmid with defined copy numbers diluted into cDNA samples prepared from wild-type mouse livers at different concentrations was used as standard sample to establish standard curve, thereby standard equation was obtained. Based on the established standard equation, the copy number of miR-shRNA transcript in each sample was calculated using the corresponding Ct value. The copy number of Rps 18 mRNA in each sample was determined in the same way using the primer pair as above. The miR-shRNA transcript copy number was normalized to that of Rps 18 mRNA in the same sample, and the resulted data was the relative miR-shRNA expression level.

### Western blot analysis

Total protein was extracted from transgenic mouse livers using Total Protein Extraction Kit (Promab, USA; Cat No.: SJ-200501) as described in the manual. Total protein samples were subjected to conventional SDS-PAGE electrophoresis, and then transferred to NC membranes (PIERCE,USA; Catalog No.:88018) using a transblot facility Mini Teans-Blot Elecreophoresis Transfer Cell (Bio-Rad, USA; Catalog No:170- 3930). Western blot (WB) was performed using Goat Anti-mouse CYP3A Antibody (Santa, USA; Cat No.: sc-30621) as primary antibody to detect CYP3A expression, and using Monoclonal Antibody to mouse GAPDH (ProMab, USA; Cat No: Mab-2005079) as primary antibody to detect the internal control GAPDH expression. The densities of Western blot bands were detected using the software Gel Pro4.0.

### Analysis of CYP3A enzymatic activity

Liver microsomes were prepared from untreated adult male mice (aged 8–10 weeks) by differential centrifugation as previously described [28], [29], and the protein concentrations of the prepared microsome suspensions were determined by Lowery method [30]. Using testosterone as the probe substrate, CYP3A activity in mouse liver microsomes was measured through isocratic HPLC as previously described [31]. Briefly, mouse liver microsome suspensions were diluted with freshly prepared NAPDH generation system to be 1 mg/mL. Testosterone dissolved into DMSO solution was added into 0.2 mL of the diluted liver microsome suspension up to a final concentration of 100 µmol/L as the probe substrate. After pre-incubation at 37°C for 5 min, 3 µL β-NADP/β-NADPH solution containing 3% NaHCO3 was added to initiate reaction. After incubation at 37°C for 30 min, 0.2 mL ice-cold acetonitrile (Sigma, Cat No.: 34998; USA) was added into the reaction mixture to terminate reaction. Thereafter, the reaction mixtures were subjected to centrifuge and the supernatants were used for HPLC analysis to detect the oxidative metabolite of probe substrate. The CYP3A activity was assessed as concentrations of the oxidative metabolite of probe substrate (6β-hydroxyl-testosterone).

### Denaturing Gradient Gel Electrophoresis (DGGE) of RT-PCR products of targeted CYP3A genes

The RT-PCR products subjected to DGGE were prepared using the degenerate primer pair with complete complementarity to all the three targeted CYP3A genes exhibiting expression in adults (CYP3A11, CYP3A41 and CYP3A44) as mentioned above, except that the reverse primer was fused 3′ to a GC-clump. The sequence of GC-clump was: 5′-CGCCCGGGGCGCGCCCCGGGCGGGGCGGGGGCACGGGGGG-3′. DGGE was performed as previously described [32], [33]. Briefly, DGGE was run through 8% (wt/vol) polyacrylamide gels of the size 16-cm×16-cm×1-mm. The gels contained a 30–50% gradient of urea and formamide increasing in the direction of electrophoresis. The 100% denaturing solution was defined as 40% (vol/vol) formamide and 7.0 M urea. Each lane was loaded with similar amount of DNA. The gels were stained with AgNO3 after electrophoresis, and then photographed using a digital camera (model D3100, Nikone, Japan). After photographing, the DNA of each band was extracted from gel, amplified by PCR using the degenerate primer pair with no GC-clump and sequenced. The digital photo of the gel was decolorized, and the intensities of DGGE bands were detected using Quantity One 4.6.2 (Bio-rad) software as described in the manual.

## Results

### shRNA design and generation of transgenic knock-down mice

CYP3A is the most abundant subfamily of CYP enzymes in mice [34]. In this study, shRNAs targeting several members of CYP3A were designed using an on-line RNAi design algorithm as described in Materials and Methods. Two shRNAs with high scores were selected, of which the sequences were 5′-AATTAAGAATGTGCTAGTGAAG-3′ (shRNA1) and 5′-AAGGTTTGCTCTCATGAATATG-3′ (shRNA2) respectively. The uniqueness of the two designed shRNAs was analyzed by BLAST. Data showed that except the first nucleotide, which was included to form the bubble structure of mature miRNA molecule, the 2–22nd nucleotides of both shRNA1 and shRNA2 exhibited complete complementarity to the mRNA sequences of CYP3A11, CYP3A16 and CYP3A44, and moreover, that of shRNA1 showed high complementarity to CYP3A41 with only one nucleotide mismatch. In addition, the two shRNAs both exhibited no homology to human CYP3A mRNA sequences, suggesting they would not silence human CYP3A expression if human CYP3A were transgenically expressed in the resulted knock-down mice.

**Figure 1:**
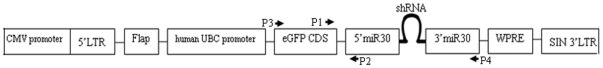
Structure of FUW-eGFP-miR-shRNA vector. The designed shRNA sequences targeting mouse CYP3A mRNAs were placed into human miR30 context downstream eGFP coding sequence (CDS). The eGFP-miR-shRNA fragments were further inserted downstream the human Ubiquitin C (UBC) promoter in the lentiviral vector FUW, as a result the eGFP-miR-shRNA fragment was under transcriptional control of human UBC promoter. Arrows P1 and P2 indicates the positions of real-time PCR primers used to detect miR-shRNA expression, and P3 and P4 the primer positions for transgenic founder mouse PCR screen.

**Figure 2:**
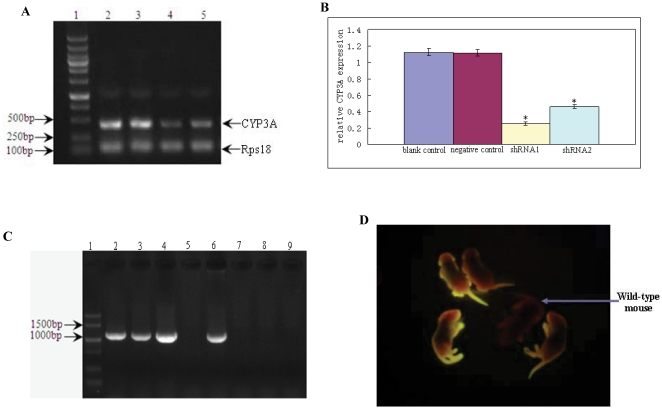
shRNA knock-down efficiency test *in vitro* and transgenic founder mouse production. A: CYP3A expression was detected by RT-PCR in hepatic cells expressing the two designed shRNAs (shRNA1 and shRNA2), shRNA targeting luciferase gene (negative control) and untreated hepatic cells (blank control) respectively. 1: DNA markers; 2: untreated hepatic cells (blank control); 3: hepatic cells infected with an unrelated shRNA targeting luciferase gene (negative control); 4: hepatic cells infected with shRNA1; 5: hepatic cells infected with shRNA2. B: The CYP3A expression level in hepatic cells expressing shRNA1 or shRNA2 was significantly lower than that of negative or blank control, and hepatic cells expressing shRNA1 exhibited the lowest CYP3A expression level. C: Transgenic mice were generated with the lentiviral vector expressing shRNA1, and four founder mice were detected to be transgenic by PCR using genomic DNA as templates. D. The four transgenic founder mice displayed fluorescence of different intensities. *: indicates statistic significance.

The two shRNA sequences were placed into miR30 context downstream eGFP CDS and thereby lentiviral vectors expressing the miR-shRNAs, named as FUW-eGFP-miR-shRNA in this article, were constructed (Fig. 1). To test the gene knock-down efficacy of the two designed shRNAs in vitro, the FUW-eGFP-miR-shRNA vectors expressing the miRNA-based versions of the two designed shRNAs (miR-shRNA1 and miR-shRNA2) and an unrelated miR-shRNA targeting luciferase gene (negative control) were packaged into lentiviral particles, and infected primarily cultured mouse hepatic cells. RT-PCR was performed to detect CYP3A expression jn infected hepatic cells using a degenerate primer pair complementary to all the three targeted CYP3As which exhibit expression in adults (CYP3A11, CYP3A41 and CYP3A44), and CYP3A16 expression was not detected because it is not expressed in adults, but predominantly in fetus [35]. Results showed that the CYP3A expression level in hepatic cells infected with shRNA1 or shRNA2 was markedly lower than that of uninfected cells (blank control) or cells infected with unrelated miR-shRNA (negative control) (Fig. 2 A, B), suggesting that the two designed shRNAs were both effective in inhibiting CYP3A expression. The hepatic cells infected with shRNA1 exhibited the lowest CYP3A expression level (Fig. 2 B), indicating that shRNA1 had a relatively higher efficiency in knock-down of target gene expression. The CYP3A expression level of negative control was comparable to that of blank control, suggesting that eGFP or unrelated miR-shRNA expression did not disturb CYP3A expression in cultured hepatic cells.

The shRNA1 was selected to generate transgenic mice using its corresponding FUW-eGFP-miR-shRNA vector by lentiviral transgenesis as previously described [26], and 4 founder mice were detected to be transgenic by PCR (Fig. 2 C). The four transgenic founder mice exhibited fluorescence of different intensities as detected by exposing to 302 nm UV light emitted from a UV light transmitter and observed through a red light filter (Fig. 2 D). The founder mouse with the strongest fluorescence was selected to breed for germline transmission. In parallel, the FUW-eGFP-miR-shRNA vector expressing a miR-shRNA targeting luciferase was also used to generate transgenic mice, which were used as negative control (data not shown).

### Transgenic miR-shRNA1 suppressed CYP3A expression in a dose-dependent manner

Because miR-shRNA was co-transcribed with eGFP in one transcript as a multi-cistron (Fig. 1), the eGFP expression level should be quantitatively proportional to that of miR-shRNA molecules. Therefore, stronger fluorescence should mean higher miR-shRNA expression level and vice versa. On the basis of this point, the four transgenic founder individuals, displaying fluorescence of different intensities, were all used to investigate the correlation between the knock-down efficiency and miR-shRNA1 expression level.

**Figure 3:**
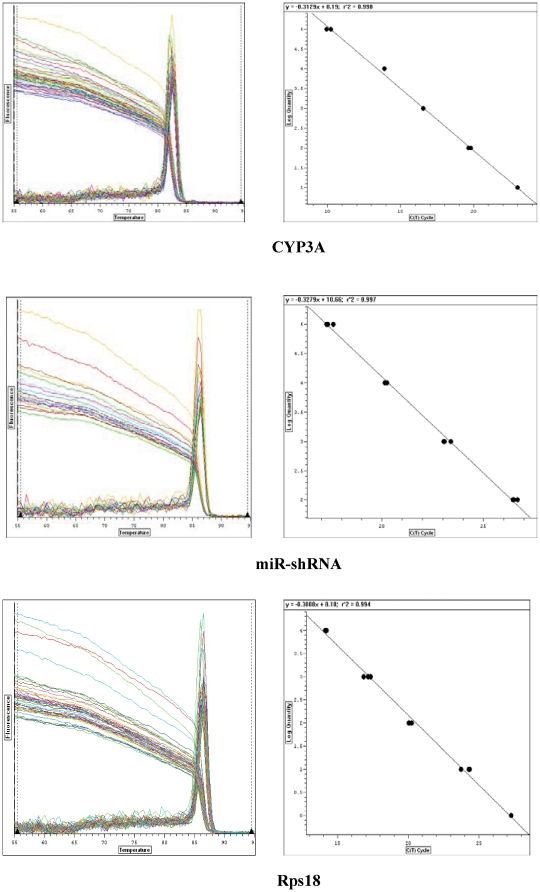
Melting and standard curves of the real-time PCR systems for CYP3A, miR-shRNA1 and Rps18. The real-time PCR was performed using cDNA samples prepared from the livers as templates. The standard equation of each real-time PCR system was y = −0.3129x+8.19, y = −0.3279x+10.66 and y = −0.3008x+8.18 respectively. The amplification efficiency (E value) of each system was 1.06, 1.18 and 1.00, and correlation coefficients (r2 values) of the standard equations were 0.998, 0.997 and 0.994 respectively.

**Figure 4:**
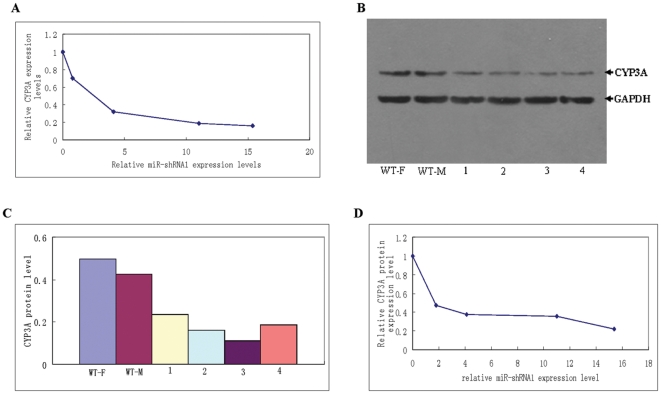
Correlation between CYP3A expression level and that of miR-shRNA1. **A**: The correlation between the relative expression level of CYP3A and that of miR-shRNA1 detected by real-time RT-PCR. The relative CYP3A expression was calculated by Pfaffl equation, and the resulted data referred to the ratio of CYP3A expression in miR-shRNA1 transgenic mice to that of wild-type individual of the same gender. The relative miR-shRNA1 expression level was detected as the copy number of miR-shRNA1 transcript normalized to that of Rps 18 mRNA in the same sample. **B**: CYP3A protein detected by Western blot in the livers of wild-type and miR-shRNA1 transgenic mice. The GAPDH protein was simultaneously detected as internal control. WT-F: female wild-type mouse; WT-M: male wild-type mouse; 1–4: the four transgenic founder individuals displaying fluorescence of different intensities. **C**: CYP3A protein levels in the four transgenic founders and wild-type mice. CYP3A protein level in each sample was calculated by comparing the WB band density of CYP3A to that of GAPDH. **D**: The correlation between relative CYP3A protein expression level and that of miR-shRNA1. Relative CYP3A protein expression level was the CYP3A protein level normalized to that of wild-type control of the same gender, which was set as 1.

The transgenic founder mouse exhibiting the strongest fluorescence was selected for breeding by mating with wild-type mice. After germline transmission was confirmed, the four transgenic founder individuals were all subjected to real-time RT-PCR analysis (Fig. 3). Because mouse CYP3A expression exhibits gender difference [35], two adult wild-type mice (one male and one female) were included as blank controls for CYP3A expression analysis, and the transgenic mice expressing an unrelated miR-shRNA also included as negative control. Results showed that in miR-shRNA1 transgenic mice, the relative CYP3A expression level, which was normalized to that of wild-type control of the same gender, was inversely correlated to the relative miR-shRNA1 expression level (Fig. 4A). Being consistent with our data for cultured hepatic cells, the transgenic mice expressing an unrelated miR-shRNA exhibited comparable CYP3A expression level to wild-type mice, indicating that expression of eGFP or an unrelated miR-shRNA did not disturb CYP3A expression in vivo either (data not shown).

The CYP3A expression in the four transgenic founder individuals was further detected by Western blot (WB). The CYP3A protein expression level in each sample was calculated by comparing the WB band density of CYP3A to that of the internal control GAPDH. Data showed that the four transgenic founder individuals, displaying fluorescence of different intensities, also exhibited different levels of CYP3A protein (Fig. 4 B, C). The relative CYP3A protein expression level, which was normalized to that of the wild-type control of the same gender, was also inversely correlated with the relative miR-shRNA1 expression levels (Fig. 4 D), being consistent with the data of real-time PCR analysis.

### Transgenic miR-shRNA1 suppressed CYP3A expression through germline transmission

**Figure 5:**
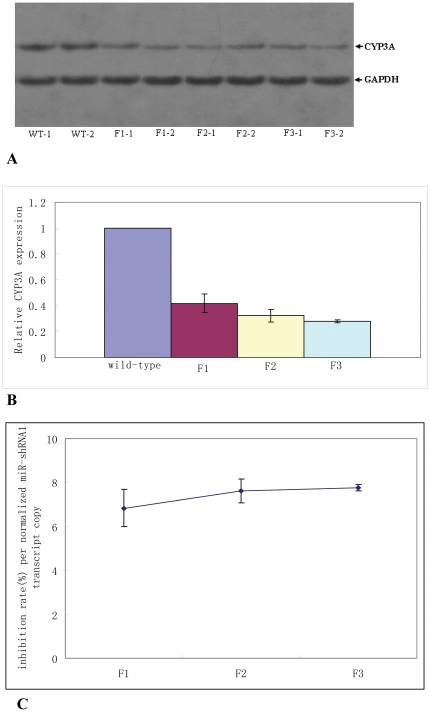
Lentiviral transgenic miR-shRNA1 suppressed CYP3A expression through germline transmission. **A**: CYP3A protein detected by WB in the two breeding mice of each generation. WT-1: female wild-type mouse; WT-2: male wild-type mouse; F1-1, F1-2: the two breeding mice of F1 generation; F2-1, F2-2: the two breeding mice of F2 generation; F3-1, F3-2: the two breeding mice of F3 generation. **B**: Relative CYP3A expression level in the breeding mice of F1–F3 generations. The relative CYP3A expression levels were calculated as described above, and the value of each generation was the average of the two breeding mice. **C**: The inhibition rate of CYP3A expression per normalized miR-shRNA1 transcript copy over generations. The value of each generation was also the average of the two breeding mice.

The transgenic founder displaying the strongest fluorescence was used for germline transmission by mating with a wild-type individual before it was used for other analysis. F1 transgenic individuals displaying variant intensities of fluorescence were obtained as a result of lentiviral integrant segregation as previously described [27], [36]. Two F1 transgenic individuals, one male and one female which displayed the strongest fluorescence among littermates of the same gender, were selected for further germline transmission by mating each other, and their offspring continued to be bred in the same way over to F3 generation as previously described [27]. The CYP3A expression levels in the breeding mice of each generation were detected by WB. Data showed that the CYP3A protein levels in the breeding mice of F1–F3 generations were all markedly lower compared to those of controls (Fig. 5 A). Moreover, the relative CYP3A protein expression level, which was normalized to that of wild-type control of the same gender, was comparable among different generations, but slightly decreased over generations (Fig. 5 B). These data suggested that transgenic miR-shRNA1 suppressed CYP3A expression in an inheritable manner.

Lentiviral transgenesis usually results in multiple integrations, and the expression level of transgene delivered by lentviral vector is closely related to lentiviral integrant number[27]. Therefore, in this study more lentiviral integrants might mean higher miR-shRNA expression level and higher knock-down efficiency as a result. For this reason, the knock-down efficiency per miR-shRNA1 molecule was calculated by comparing the inhibition rate of CYP3A expression to the relative miR-shRNA1 expression level (the miR-shRNA1 transcript copy number normalized to that of Rps 18 mRNA) in one individual. The inhibition rate of CYP3A expression was calculated as the following: inhibition rate = (1−CYP3A expression normalized to wild-type individual)×100%. The inhibition rate per normalized miR-shRNA1 transcript copy of each generation was the average value of the two breeding mice. As shown in Fig. 5 C, the inhibition rate per normalized miR-shRNA1 transcript copy remained relatively constant, but slightly increased over generations (Fig. 5 C), further suggesting that lentiviral transgenic miR-shRNA1 suppressed CYP3A expression in an inheritable manner.

Because mouse CYP3A expression exhibits gender difference, to investigate whether the knock-down efficacy observed in this study also exhibited gender difference, we compared the inhibition rate of CYP3A protein expression between male and female transgenic mice using the data derived from the breeding mice of F1–F3 generations. Paired T-test was performed using the data of the two breeding mice of each generation as a pair. Result showed that no remarkable difference was observed for the inhibition rates between male and female mice (P = 0.9192). Actually, female and male transgenic mice exhibited a very similar inhibition rate (0.64±0.096 vs 0.65±0.076), indicating that the transgenic miR-shRNA1 reduced CYP3A expression to a defined degree regardless of gender.

### Transgenic miR-shRNA1 remarkably reduced CYP3A enzymatic activity

**Figure 6:**
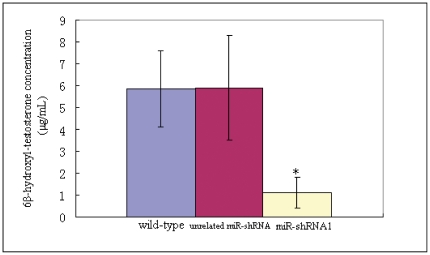
CYP3A enzymatic activity analysis. Five adult male miR-shRNA1 transgenic mice, unrelated miR-shRNA transgenic mice and wild-type mice were subjected to CYP3A enzymatic activity analysis in parallel. The ages of used mice were 8–10 weeks. Analysis was performed using liver microsome systems prepared from each group, and testosterone was added into the systems up to a defined concentration as the probe substrate. The concentration of the oxidative metabolite of probe substrate (6β-hydroxyl-testosterone) was detected by HPLC analysis. Wild-type: wild-type mice of FVBN strain; unrelated miR-shRNA: transgenic mice expressing a miR-shRNA targeting luciferase; miR-shRNA1: transgenic mice expressing the miR-shRNA1 targeting CYP3A. * indicates statistic significance.

To further investigate the knock-down efficacy of transgenic miR-shRNA1, CYP3A enzymatic activity in knock-down mice was measured. Untreated adult male miR-shRNA1 transgenic, unrelated miR-shRNA transgenic and wild-type mice of 8 week age were subjected to CYP3A enzymatic analysis in parallel (five mice/each group). To measure the CYP3A activity, testosterone was added into liver microsome suspensions up to a defined concentration (100 µmol/L) as probe substrate. Data showed that the concentrations (µg/mL) of the oxidative metabolite of testosterone (6β-hydroxyl-testosterone) in the liver microsome suspensions of miR-shRNA1 transgenic mice were significantly lower than those of wild-type mice (1.11±0.71 vs 5.85±1.74, P = 0.0005) or transgenic mice expressing an unrelated miR-shRNA (1.11±0.71 vs 5.9±2.4, P = 0.00047) (Fig. 6), indicating that the CYP3A enzymatic activity was markedly reduced in the knock-down mice. In addition, the 6β-hydroxyl-testosterone concentrations in liver microsome suspensions were comparable between unrelated miR-shRNA transgenic and wild-type mice (5.85±1.74 vs 5.9±2.4, P = 0.9786) (Fig. 6), further indicating that the expression of eGFP or an unrelated miR-shRNA had no effect on CYP3A enzymatic activity, which was consistent with the data for CYP3A expression analysis.

### The targeted CYP3A genes in knock-down mice exhibited similar characteristics in RT-PCR DGGE analysis to those in controls

**Figure 7:**
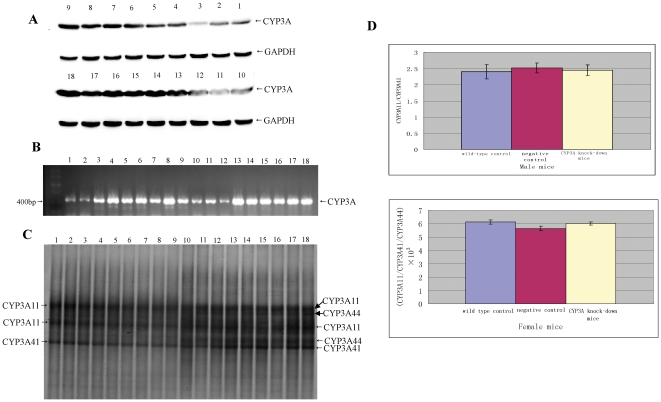
DGGE analysis of the mixed RT-PCR products of targeted CYP3A genes. The mixed RT-PCR products were prepared using a degenerate primer pair from randomly selected knock-down and control mice of 5-week age, and subjected to DGGE with similar amount of DNA loaded into each lane. A: Overall CYP3A protein detected by WB in knock-down, wild-type (blank control) and unrelated miR-shRNA transgenic (negative control) mice. B: The mixed RT-PCR products prepared from knock-down and control mice. C: The mixed RT-PCR products were subjected to DGGE. D: The ratio of targeted CYP3A expression levels, estimated by comparing the corresponding DGGE band intensities of each gene. 1∼3: male knock-down mice; 4∼6: male unrelated miR-shRNA transgenic mice; 7∼9: male wild-type mice; 10∼12: female knock-down mice; 13∼15: female unrelated miR-shRNA transgenic mice; 16∼18: female wild-type mice.

To address whether the three targeted CYP3A genes which exhibit expression in adults were all suppressed in the knock-down mice, mixed RT-PCR products of the three genes were prepared using the degenerate primer pair as described above from randomly selected knock-down mice and control (wild-type and unrelated miR-shRNA transgenic) mice at 5-week age of both genders (Fig. 7 B). The mice of 5-week age were used because it had been reported that at this age the three CYP3As were expressed in both male and female [33]. The selected knock-down mice exhibited markedly reduced overall CYP3A protein level compared to controls of the same gender (Fig. 7 A). The mixed RT-PCR products were subjected to DGGE analysis, a regular method designed to separate homologous DNA fragments of the same length with base-pair difference [33]. Data showed that the knock-down mice exhibited almost the same DGGE band profile as controls of the same gender (Fig. 7 C). In female group, CYP3A11, CYP3A41 and CYP3A44 were all detected, while in male group CYP3A11 and CYP3A41 detected. The ratios of the RT-PCR products of different CYP3As (CYP3A11/CYP3A41 in male group and CYP3A11/CYP3A41/CYP3A44 in female), which were estimated by comparing the corresponding DGGE band intensities of each gene, were comparable between knock-down and control mice of the same gender (Fig. 7 D). These data indirectly indicated that the transcriptional levels of the three targeted CYP3As were reduced to a similar extent in knock-down mice.

## Discussion

CYP3A is the most abundant CYP enzyme subfamily both in human and mammalian animals, which are involved in the metabolism of more than 50% of clinically available drugs for humans. Therefore, CYP3A is the prior target to disrupt for generation of animals lacking or with largely reduced endogenous CYP expression. In this study, we designed a microRNA-based shRNA (miR-shRNA) molecule simultaneously targeting four members of mouse CYP3A subfamily: CYP3A11, CYP3A16, CYP3A41 and CYP3A44, of which the sequence exhibited no homology to human CYP3A genes. Knock-down mice with markedly reduced CYP3A expression were generated by transgenic expression of the designed miR-shRNA driven by a PolII (human Ubiquin C) promoter. Using the four transgenic founder individuals exhibiting different levels of miR-shRNA expression, the correlation between the knock-down efficiency and miR-shRNA expression level was directly investigated. Our data showed that the transgenic miR-shRNA molecule suppressed CYP3A expression in a dose-dependent manner in vivo, suggesting that to generate knock-down animals with efficient inhibition of target gene expression using this system, in addition to design potent shRNA molecules, establishment of a transgenic line with high miR-shRNA expression level is also necessary.

Germline transmission of knock-down efficacy is the perquisite to establish a knock-down animal line lacking or with largely reduced target gene expression. Our data showed that the CYP3A expression levels in the transgenic offspring of F1–F3 generations, which were all markedly lower than those of wild-type mice, were comparable and moreover slightly decreased over generations. In addition, the knock-down efficiency per miR-shRNA transcript copy also slightly increased over generations. These results confirmed that the lentiviral transgenic miR-shRNA suppressed CYP3A expression in an inheritable manner, suggesting that the lentiviral integrants expressing miR-shRNA were not subjected to silence through germline transmission, which was consistent with our previous data of lentviral transgene expression analysis [27]. The slight increase of knock-down efficiency over generation may be due to the breeding strategy used in this study, through which the lentiviral integrants exhibiting higher miR-shRNA expression level were preferred to be transmitted through germline. On this basis, this work provided a strategy to establish a transgenic line with an inheritable and efficient knock-down of target gene expression using lentiviral transgenic animals.

The knock-down efficacy observed in this study was further confirmed by CYP3A enzymatic activity analysis. The average CYP3A enzymatic activity in liver microsome suspensions of knock-down mice was reduced to about twenty percent of that of wild-type mice or transgenic mice expressing unrelated miR-shRNA. The degree of CYP3A enzymatic activity reduction was slightly higher than that of CYP3A protein reduction. One reason for this may be that different members of CYP3A subfamily may exhibits different activity or reaction kinetics in the metabolism of substrates, which has already been observed in human [37]. Therefore, a certain degree of CYP3A protein reduction may result in a higher degree of CYP3A enzymatic activity reduction if the inhibited CYP3A enzymes exhibited a relatively higher activity. Being consistent with the data of CYP3A expression analysis, the expression of eGFP or unrelated miR-shRNA did not affect mouse CYP3A enzymatic activity either, indicating that the reduction of CYP3A enzymatic activity in knock-down mice was exactly mediated by the designed transgenic miR-shRNA molecule.

Due to the high homology of the three targeted CYP3A genes which exhibit expression in adults (CYP3A11, CYP3A41 and CYP3A44), we failed to design gene-specific primer pairs or Taqman probes to detect each gene expression quantitatively one by one. Besides, semi-quantitative analysis of each gene expression by WB was not achieved either due to the lack of antibodies targeting the three CYPs individually. By DGGE of the mixed RT-PCR products of the three CYP3As prepared using a degenerate primer pair, we separated the RT-PCR products of the three genes. Data showed that the mixed RT-PCR products of knock-down mice exhibited almost the same DGGE band profile to those of control mice, and the ratios of RT-PCR products of the three CYP3As were comparable between knock-down and control mice. This result provided indirect evidence that the transcriptional levels of the targeted CYP3As may be suppressed to a similar extent in knock-down mice, even though the designed transgenic miR-shRNA molecule had one nucleotide mismatch with CYP3A41. However, this indirect evidence did not mean that the targeted CYP3As were all actually reduced to a similar extent on protein level. By DGGE analysis, CYP3A44 were not detected in male group, which was not consistent with a previous report [33] demonstrating that both CYP3A41 and CYP3A44 were expressed in male C57BL6 mice of 5-week age. This inconsistence may be due to the fact that a different mouse strain (FVBN) was used in this work, and the CYP3A44 expression level in male FVBN mice of 5-week age may be too low to be detected by DGGE analysis.

Generation of knock-down animals lacking or with largely reduced endogenous CYP3A expression is the ultimate goal of this study. In this study, we designed a miR-shRNA simultaneously targeting four members of CYP3A subfamily, but the remaining three CYP3A members (CYP3A25, CYP3A57 and CYP3A59) were not targeted, which may be the main reason for the residual level of CYP3A protein or CYP3A activity. Among the remaining three CYP3A members, CYP3A25 is considered to be the second most abundant CYP enzyme in mice which exhibits a constitutive expression with no gender or organ difference. Future work can be focused on the knock-down of the remaining three CYP3A members by the same strategy described in this work. Sequence comparison indicates that the three mouse CYP3A genes exhibit homology of a similar degree to that of the four CYP3A members targeted in this study. Thus, one miR-shRNA can be designed to disrupt the three CYP3A gene expressions. On this basis, a “bigenic” line simultaneously expressing two designed miR-shRNA molecules can be established, and a knock-down mouse line lacking or with largely reduced expression of all the CYP3A genes can be obtained. In addition, considering that RNAi is a highly conserved mechanism, and lentviral transgenesis is an extremely efficient method for transgenic animal production which has been proved to be applicable to a variety of mammalian species including non-human primates [26], [38]–[41], the strategy described in this study can be further applied to other mammalian species, especially those species of great value for drug development which are highly refractory to traditional ES cell-based gene knock-out technology, such as dog, minipig and non-human primates. However, to use this system for these purposes, one point should be taken into consideration that lentiviral transgenic miR-shRNA can suppress endogenous CYP expression largely, but not entirely. For many pharmacokinetic or pharmacodynamic studies to evaluate drugs or investigate the relation of a particular human CYP to a specific drug toxicity or inefficacy, low residual level of endogenous CYP may not be a big hurdle. But for those experiments requiring no endogenous CYP expression, this system is not an optimal choice. Another limitation of this system is multiple integrations of lentiviral transgene, which limits to establish homozygous transgenic line with stable transgene expression. However, our previous data [27] indicated that, by selecting the individuals exhibiting the strongest transgene expression as the breeding animals for each generation, a “transgenic colony” with relatively constant and high transgene expression can be established after several generations of breeding in this way consecutively. Therefore, on this basis a homozygous line was not required. In addition, because this system contains a marker gene being co-transcribed along with miR-shRNA molecules, it would be convenient to use the transgenic animals derived from this system for this purpose, which was also suggested by the data of this work.
